# Neonatal and infant diagnostic HIV‐PCR uptake and associations during three sequential policy periods in Cape Town, South Africa: a longitudinal analysis

**DOI:** 10.1002/jia2.25212

**Published:** 2018-11-27

**Authors:** Emma Kalk, Max Kroon, Andrew Boulle, Meg Osler, Jonathan Euvrard, Kathryn Stinson, Venessa Timmerman, Mary‐Ann Davies

**Affiliations:** ^1^ Centre for Infectious Disease Epidemiology & Research School of Public Health & Family Medicine University of Cape Town Cape Town South Africa; ^2^ Department of Paediatrics Mowbray Maternity Hospital University of Cape Town Cape Town South Africa; ^3^ Health Impact Assessment Provincial Government of the Western Cape Cape Town South Africa

**Keywords:** HIV, vertical transmission, early infant diagnosis, birth HIV‐PCR, guideline implementation, risk factors for HIV transmission, maternal and child health

## Abstract

**Introduction:**

To strengthen the early infant diagnosis (EID) programmes and timeously identify and treat HIV‐infected infants, birth HIV‐PCR for some/all infants has been recommended in the Western Cape, South Africa since 2014. Operational data on the implementation of such programmes in low‐ and middle‐income countries are limited.

**Methods:**

Utilizing the electronic records platform at primary care facilities, we developed an electronic register which consolidated obstetric and HIV‐related data, allowing us to track a cohort of HIV‐infected/exposed mother/infant dyads longitudinally from antenatal care through delivery to infant HIV‐PCR. We assessed guideline implementation and impact on EID of three sequential EID policies in a referral chain of facilities in Cape Town (primary‐tertiary care). Birth HIV‐PCR was indicated in period 1 if symptomatic; period 2 if meeting high‐risk criteria for transmission; and period 3 for all HIV‐exposed neonates.

**Results:**

We enrolled 2012 HIV‐exposed infants; 89.2% had at least one HIV‐PCR at any point. The majority of birth tests were performed in hospital versus primary care regardless of policy period. Almost half of all infants (47.9%) had at least one high‐risk criterion for vertical infection; of these, 39.7% had a birth test. Infants with more risk factors were more likely to have birth EID. Receipt of a birth HIV‐PCR significantly reduced the likelihood of receiving a follow‐up test at six to ten weeks, even after adjusting for potential confounders (aOR 0.18 (0.12 to 0.26)). The proportion of infants tested at six to ten weeks old dropped from 92.9% (period 1) to 80.2% in period 3 and those receiving birth HIV‐PCR increased, peaking at 67.4% during period 3. The proportion of positive birth tests was highest (2.9%) when birth tests were restricted to infants meeting high‐risk criteria, with a low proportion positive for the first time at six to ten weeks. During period 3, the proportion positive at six to ten weeks was high (2.4%), highlighting the importance of follow‐up to detect intrapartum and early postpartum infections.

**Conclusions:**

Over all policy periods, EID guidelines were incompletely implemented across all levels of care but especially in primary care. Birth HIV‐PCR reduced return for follow‐up testing, such follow‐up testing is critical for the effectiveness of the programme.

## 1 Introduction

The introduction of birth HIV‐PCR for HIV‐exposed infants as part of early infant diagnosis (EID) has been promoted as a means of maximizing the effectiveness of EID programmes by increasing testing coverage, and identifying *in utero* HIV‐infected neonates allowing early initiation of antiretroviral therapy (ART) 1. The World Health Organization (WHO) included a conditional recommendation in the 2015 vertical transmission of HIV prevention (VTP) guidelines for a diagnostic nucleic acid test around birth (zero to two days), in addition to routine HIV testing at four to six weeks, either for all HIV‐exposed infants or for those determined to be at high risk of vertical infection (targeted birth testing) 2.

It has been suggested that birth HIV‐PCR will address some of the challenges associated with achieving high coverage of EID at the four‐ to six‐week testing time‐point which result in EID gaps in many programmes in low‐ and middle‐income countries (LMIC). In 2015 operational delays, with attrition at all points along the EID continuum, resulted in only an estimated 51% of HIV‐exposed infants receiving an HIV‐PCR before two months old in the WHO 21 priority countries 3. Even fewer initiated ART 4. Models suggest that the addition of birth HIV‐PCR to testing algorithms would increase the number of infants diagnosed with HIV and therefore life‐years saved 5, 6.

Since birth HIV‐PCR cannot detect infections due to intrapartum and postnatal transmission, a subsequent early HIV test is still required (e.g. at six to ten weeks). Indeed, models suggest that the clinical and financial benefits of adding birth HIV‐PCR may be eliminated if loss to follow‐up (LTFU) at subsequent testing times exceeds 37% 5. Given the cost implications of additional nucleic acid assays and the often poor implementation of current EID guidelines, there is debate as to the optimal number, and timing, of diagnostic tests 7, 8. This has prompted calls for operational data to evaluate birth HIV testing in routine settings in LMIC 7.

In 2015, the South African (SA) Department of Health introduced birth HIV‐PCR for all HIV‐exposed infants, with a second test at ten weeks old, becoming the first national programme to do so in sub‐Saharan Africa 9. In the Western Cape Province targeted birth testing had been policy since August 2014.

We aimed to examine the uptake of infant HIV testing under three different EID policies in a referral chain of facilities in Cape Town (from primary care to district, secondary, and tertiary level hospitals) using prospective, longitudinally collected individual patient data. We assessed adherence to provincial EID guidelines, the yield of HIV‐PCR at birth at the sites, and the impact on presentation for follow‐up testing.

## 2 Methods

This study formed part of an implementation science project aimed at assessing VTP coverage and effectiveness with an active surveillance system in the form of an electronic register (e‐register). Using the digitized medical record platform in primary care facilities, the e‐register prospectively consolidated routinely collected clinical data from paper‐based obstetric and HIV registers. HIV‐associated laboratory results and ART prescriptions for each participant were integrated.

### 2.1 Setting and participants

We included routinely collected data from birth onwards of HIV‐exposed live infants of women entered in the e‐register. The women had attended antenatal care (ANC) and/or delivered at Mitchell's Plain Midwife Obstetric Unit (MPMOU), an urban primary care facility and its referral centres in Cape Town, SA. Between February 2014 and December 2015, all women presenting to MPMOU for ANC with an expected or actual delivery date before June 2016, regardless of HIV status, were enrolled. Women who presented for the first time in labour (i.e. received no ANC) were enrolled until June 2016. Deliveries were followed through December 2016 and HIV‐PCR results collected until June 2017 for all participants. Uncomplicated vaginal deliveries were managed by midwives at MPMOU. Approximately half of all women were referred to hospital during pregnancy or intrapartum. MPMOU referred to a District Hospital (with operating theatres), a Level 2 Maternity Hospital (neonatal Intensive Care Unit (ICU)), and a tertiary hospital (adult and neonatal ICU), based on standardized criteria.

WHO Option B+ was the VTP policy in place over the study period 10, 11, 12. Women of negative/unknown HIV status were offered HIV testing (rapid assay) at their first antenatal visit, during the third trimester, and during labour/immediately postpartum. HIV‐infected women initiated life‐long ART. HIV‐exposed infants received six to twelve weeks of daily nevirapine (NVP) depending on whether the mother had received ≥8 weeks of ART before delivery or not. In 2015, these guidelines were amended so that neonates at low risk of vertical transmission received six weeks of NVP; for high‐risk infants NVP was extended to twelve weeks with zidovudine added for the first six weeks 10.

### 2.2 EID testing periods

The Western Cape EID guidelines changed twice during the course of the study, leading to three EID policy periods 10, 11, 12. During *period 1* (May 2013‐July 2014), birth HIV‐PCR was offered where there was clinical suspicion of HIV infection, in addition to the routine six‐week test 11. During *period 2* (August 2014‐November 2015), additional birth HIV‐PCR was indicated in the presence of defined high‐risk criteria for vertical transmission; the six‐week test remained routine (Table 1)^12^. During *period 3* (from December 2015) birth HIV‐PCR was indicated for *all* HIV‐exposed infants regardless of transmission risk, with an additional test at ten weeks old in place of six weeks 10.

**Table 1:** High‐risk criteria for vertical transmission of HIV^12^

Maternal factors	Infant factors
Diagnosed with HIV after 28 weeks of gestation	Born before 37 completed weeks of gestation
HIV seroconversion during pregnancy	Birthweight below 2500 g
Less than 12 weeks of ART before delivery	
Plasma viral load greater than 1000 copies/mL	

### 2.3 Procedures and measurements

The e‐register provided a single longitudinal record for each mother‐infant dyad and included HIV testing and ART history from first antenatal visit through to infant HIV‐PCR. A woman was considered to seroconvert during pregnancy if she tested positive for HIV after an initial negative antenatal result. Results of maternal viral load testing performed during pregnancy or within two weeks of delivery were included. No study‐related investigations were included in the analyses; all test results in the e‐register are from those conducted routinely by health care staff. Infant HIV‐PCR were deemed “birth tests” if they occurred within seven days of birth and as “six‐ to ten‐week tests” if they occurred between four and fourteen weeks old. This window included the follow‐up testing time‐points across all policy periods. A positive birth HIV‐PCR indicated *in utero* infection. Intrapartum transmission was defined as a negative HIV‐PCR at birth but subsequently positive in the absence of breast‐feeding. Postpartum infection was defined as HIV‐PCR negative at birth and six to ten weeks old but subsequently positive in the presence of breast‐feeding 4.

### 2.4 Linking maternal and infant data

According to standard procedures at MPMOU, key delivery elements were entered by midwives onto a digitized medical records system generating an infant identifier which linked the mother‐infant pair. A linked infant folder number was similarly generated at the hospitals. This linkage was distinct from the e‐register. The sample was limited to those mother‐infant pairs that could be linked (96.6% live‐born HIV‐exposed infants in the cohort).

### 2.5 Analysis

Analysis was performed using STATA v.15.0 (Stata Corporation, College Station, TX, USA). Continuous variables were summarized using means and confidence intervals (CI) or medians and interquartile ranges (IQR) for normally and non‐normally distributed variables respectively. Categorical variables were described using proportions, and frequency tables used for comparison. Significance was tested using a two‐sample *t*‐test or Wilcoxon rank‐sum test depending on the distribution for numerical data and the *χ* ^2^ test or Fishers Exact test for categorical data. The predictors of follow‐up HIV‐PCR were assessed using logistic regression. Multivariate models were fitted including known or suspected risk factors for the primary outcomes.

### 2.6 Ethics

The study was approved by the University of Cape Town Human Research Ethics Committee and the Provincial Government of the Western Cape Department of Health Research Committee.

A waiver of consent was granted for the e‐register since the data were collected as part of routine care by the health services and entered on the provincial medical records platform falling within formal protection policies. Assessments of guideline implementation were presented to facilities during the course of the study to reduce missed opportunities for infant testing.

## 3 Results

**Figure 1:**
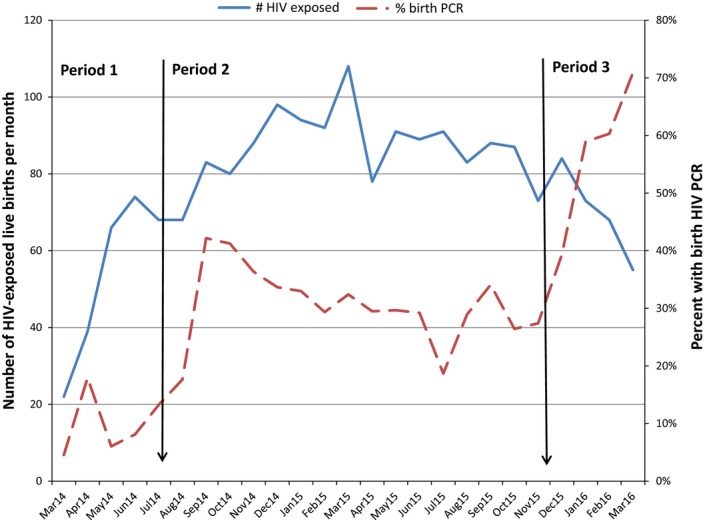
The number of HIV‐exposed infants and the percentage receiving birth HIV‐PCR during the three policy periods Change in EID policy is indicated by the black arrows. Generation of infant numbers at MPMOU was poor at the start of period 1, affecting linkage of infants and their mothers. Some exposed neonates were therefore not included. Few infants were born to enrolled women after March 2016 (antenatal recruitment having ceased in December 2015).

We included 2012 HIV‐exposed infants, 272 (13.5%) born in period 1, 1391 (69.1%) in period 2, and 349 (17.4%) in period 3. The proportion of infants receiving a birth HIV‐PCR increased over the study period with rapid increases following guideline changes (Figure 1). Median age at birth HIV‐PCR was zero days (IQR 0 to 1.4).

### 3.1 Risk factors

Over the study period, 47.9% of mother‐infant pairs had at least one risk factor for vertical HIV transmission (Table 2). Viral load was available for 1346 women (66.9%) and was >1000 copies/mL in 201 (14.9%). The number of risk factors per infant remained stable over time but the proportion of infants whose mothers were diagnosed with HIV during pregnancy (including seroconversion) decreased from 3.7% in period 1 to 1.4% in period 3. Similarly, the proportion of women who received <12 weeks of ART prior to delivery dropped: 28.3% in period 1, 21.5% in period 2 and 12.6% in period 3. Time on ART prior to delivery increased from a median of 20.1 weeks (IQR 10.6 to 39.9) to 21.1 weeks (12.1 to 85.7) and 25.3 weeks (12.6 to 88.9), in periods 1 to 3 respectively. By mid‐2016, over 40% of HIV‐infected women had conceived on ART. The percentage of infants born <37 weeks of gestation or with birthweight <2500 g remained stable over periods 2 and 3 (6.7% and 5.2%, and 14.1% and 12.3% respectively) but was lower in period 1 (1.5% and 7.0%).

**Table 2:** The proportion of infants per high‐risk criterion who received a birth HIV‐PCR per policy period

Risk factor	Total n = 2012			Period 1 n = 272			Period 2 n = 1391			Period 3 n = 349		
	Total n (%)^b^	Birth n (%)^c^	*p*	Total n (%)^b^	Birth n (%)^c^	*p*	Total n (%)^b^	Birth n (%)^c^	*p*	Total n (%)^b^	Birth n (%)^c^	*p*
HIV diagnosis during pregnancy												
Yes	122 (6.1)	52 (42.6)	0.02	10 (3.7)	4 (40)	0.012^a^	104 (7.5)	45 (43.3)	0.004	8 (2.3)	3 (37.5)	0.272^a^
No	1890 (93.9)	615 (32.5)		262 (96.3)	24 (9.2)		1287 (92.5)	383 (29.8)		341 (97.7)	208 (61)	
Antenatal care												
None	103 (5.1)	48 (46.6)	0.003	13 (4.8)	4 (30.8)	0.034^a^	66 (4.74)	35 (53.0)	<0.0001	24 (6.9)^d^	9 (37.5)	0.017
≥1 visit	1909 (94.9)	619 (32.4)		259 (95.2)	24 (9.3)		1325 (95.3)	393 (29.7)		325 (93.1)	202 (65.2)	
Seroconversion during pregnancy												
Yes	31 (1.5)	15 (48.7)	0.069	3 (1.1)	1 (33.3)	0.279^a^	23 (1.7)	12 (52.2)	0.025	5 (1.4)	2 (40)	0.388^a^
No	1981 (98.5)	652 (32.9)		269 (98.9)	27 (10.0)		1368 (98.4)	416 (30.4)		344 (98.6)	209 (60.8)	
Duration of ART												
<12 weeks	420 (20.9)	191 (45.5)	<0.0001	77 (28.3)	20 (26.0)	<0.0001	299 (21.5)	147 (49.12)	<0.0001	44 (12.6)	24 (54.6)	0.391
>12 weeks	1592 (79.1)	476 (29.9)		195 (71.7)	8 (4.1)		1092 (78.5)	281 (25.7)		305 (87.4)	187 (61.3)	
Viral load n = 1346												
>1000 copies/mL	201 (10.0)	105 (52.2)	<0.0001	7 (2.6)	2 (28.6)	0.076^a^	145 (10.4)	80 (55.6)	<0.0001	49 (14)	23 (46.9)	0.037
<1000 copies/mL	1811 (30.0)	562 (31.0)		275 (97.4)	26 (9.8)		1249 (89.6)	348 (27.9)		300 (86)	188 (62. 7)	
Gestational age (weeks) n = 1898												
<37 weeks	115 (6.1)	83 (72.2)	<0.0001	4 (1.6)	1 (25)	0.350^a^	93 (7.0)	68 (73.1)	<0.0001	18 (5.2)	14 (77.8)	0.139^a^
>37 weeks	1783 (93.9)	523 (29.3)		252 (98.4)	25 (9.9)		1228 (93)	321 (26.1)		303 (86.8)	177 (58.4)	
Birthweight (grams) n = 1928												
<2500 g	260 (13.5)	159 (32.9)	<0.0001	19 (7.6)	3 (15.8)	0.290^a^	196 (14.6)	125 (63.8)	<0.0001	45 (13.4)	31 (68.9)	0.174
>2500 g	1668 (86.5)	476 (28.5)		232 (92.4)	22 (9.58)		1144 (85.4)	284 (24.8)		292 (86.7)	170 (58.2)	

ART, antiretroviral therapy; Viral load, Viral load recorded during pregnancy or within two weeks of delivery; Gestational age, gestational age at birth as recorded in the labour ward delivery register; Birthweight <2500 g meets the clinical definition of low birthweight. ^a^Statistical test: Fisher Exact test; otherwise *χ* ^2^ test used to assess the difference between the proportions with and without birth HIV‐PCR per high‐risk criterion. ^b^The proportion of infants with a given risk factor. ^c^The proportion of infants with the risk factor who received birth HIV‐PCR. ^d^The proportion of women who received no antenatal care is slightly higher during period 3 as we continued to enrol “unbooked” women to June 2016.

The proportion of infants receiving a birth HIV‐PCR increased from 11.8% in period 1, to 34.5% in period 2 and 67.4% in period 3. Over the study period, 39.7% of neonates with one or more high‐risk criterion received birth EID. The association between having a high‐risk criterion and receiving a birth test was strongest in period 2.

### 3.2 Facility of birth

The majority of the infants (41.7%) were born at MPMOU with the numbers dropping as the level of care increased (Table 3). At all sites, the proportion of HIV‐exposed infants receiving birth HIV‐PCR increased with the guideline changes. Most of the birth tests were performed in hospital and not at the primary care site, even in period 3 where coverage of birth testing at the primary care site was only 35%.

**Table 3:** The proportion of infants with birth HIV‐PCR born in each facility per policy period

Facility	Total n = 2012		Period 1 n = 272		Period 2 n = 1391		Period 3 n = 349	
	Total n (%)	Birth n (%)^a^	Total n (%)	Birth n (%)^a^	Total n (%)	Birth n (%)^a^	Total n (%)	Birth n (%)^a^
MPMOU	838 (41.7)	150 (17.9)	126 (46.3)	6 (4.8)	548 (39.4)	87 (15.9)	164 (47.0)	57 (34.8)
MPDH (level 1)	710 (35.3)	301 (42.4)	83 (30.5)	1 (1.2)	520 (37.8)	207 (39.8)	107 (30.7)	93 (86.9)
MMH (level 2)	343 (17.1)	141 (41.1)	59 (21.7)	20 (33.9)	235 (16.9)	82 (34.9)	49 (14.0)	39 (79.6)
GSH (level 3)	98 (4.9)	61 (62.2)	3 (1.1)	1 (33.3)	75 (5.4)	46 (61.3)	20 (5.7)	14 (70)
Other	23 (1.1)	14 (60.9)	1	0	13 (0.9)	6 (46.4)	9 (2.6)	8 (88.9)
All facilities	2012	667 (33.2)	272 (13.5)	28 (10.3)	1391 (69.1)	428 (30.8)	349 (17.4)	211 (60.5)

GSH, Groote Schuur Hospital (level 3); MPMOU, Mitchell's Plain Midwife Obstetric Unit; MPDH, Mitchell's Plain District Hospital (level 1); MMH, Mowbray Maternity Hospital (level 2); Other, MOUs and hospitals in the Western Cape outside the study facilities. ^a^The proportion of infants born at each facility who received birth HIV‐PCR.

### 3.3 Timing of HIV‐PCR and follow‐up testing

Most (89.2%; 95% CI 87.7% to 90.5%) HIV‐exposed infants had at least one HIV‐PCR, 88.1% (95% CI 87.0 to 90.0) at six to ten weeks (Table 4). This dropped from 93.2% (95% CI 89.3 to 96.1) of infants having HIV‐PCRs at six weeks in period 1 to 80.2% (95% CI 75.3 to 84.5) in period 3. No HIV‐PCR result could be found for 218 (10.8%) infants; death was confirmed in three. Among 656 infants who tested HIV‐PCR negative *at birth*, 526 had a follow‐up HIV‐PCR per guideline recommendations (80.2%).

**Table 4:** Timing of HIV‐PCR by policy period and HIV transmission

	Total n = 2012	Period 1 n = 272	Period 2 n = 1391	Period 3 n = 349
At least one HIV‐PCR n (%; 95% CI)	1794 (89.2; 87.7 to 90.5)	238 (87.5; 83.0 to 91.2)	1242 (89.3; 87.5 to 90.9)	313 (89.7; 86.0 to 92.7)
Single HIV‐PCR only^a^ n (%; 95% CI)	1149 (64.0; 61.8 to 66.3)	182 (76.5; 70.6 to 81.7)	829 (66.7; 64.0 to 69.4)	138 (44.1; 38.5 to 49.8)
Age at first PCR, weeks median (IQR)	6.0 (0.1 to 6.6)	6.3 (6.0 to 6.9)	6.1 (0.1 to 6.6)	0.1 (0 to 6.1)
Birth HIV‐PCR present n (%;95% CI)	667 (37.2; 34.9 to 39.5)	28 (11.8;0.1 to 16.6)	428 (34.5;31.8 to 37.2)	211 (67.4;61.9 to 72.8)
Six to ten weeks HIV‐PCR present^a^ n (%; 95% CI)	1578 (88.6;87 to 90)	221 (93.2;89.3 to 96.1)	1106 (89.8;88 to 91.5)	251 (80.2;75.3 to 84.5)
Follow‐up HIV‐PCR ever after birth test^a^ n (%)	526 (78.8; 75.6 to 81.9)	21 (75;55.1 to 89.3)	337 (78.7;74.6 to 82.5)	168 (79.6; 73.5 to 84.8)
Age F/U HIV‐PCR^a^, weeks median (IQR)	6.5 (6.1 to 8.3)	6.3 (6.1 to 6.6)	6.4 (6.0 to 7.1)	8.6 (6.4 to 10.7)
HIV‐PCR positive ever n(%; 95% CI)	32 (1.8; 1.2 to 2.5)	1 (0.4;0.01 to 2.3)	25 (2.0; 1.3 to 3.0)	6 (1.9; 0.7 to 4.1)
Overall transmission rate	1.8	0.4	2.0	1.9
*In utero* transmission rate (95% CI)	1.7 (0.8 to 2.9)	3.6 (0.1 to 19)	2.3 (1.1 to 4.3)	0
Transmission rate at six to ten weeks^a^ (95% CI)	0.6 (0.3 to 1.0)	0	0.5 (0.1 to 1.1)	2.3 (0.9 to 5.1)
Median (IQR) age HIV‐PCR positive, weeks	6.4 (0.4 to 40)	0.1	6.0 (0.1 to 40.1)	6.8 (6.4 to 12)

a Denominator excludes those with positive birth HIV‐PCR.

### 3.4 HIV transmission

HIV transmission in all infants for whom an HIV‐PCR result was available was 1.8% (32/1794). This included *in utero*, intrapartum and postpartum infections (Table 4). The median age at HIV diagnosis was 6.4 weeks. The *in utero* transmission rate (i.e. proportion of infants with birth tests in whom HIV infection was detectable at birth) was 3.6% in period 1 (n = 1) and 2.3% in period 2. There were no positive birth HIV‐PCR results in period 3. Overall, 34.4% of all HIV infections were identified at birth – the proportion being highest in period 2 (40%). In period 3 100% of HIV infections were detected at six weeks or later.

Of all birth HIV‐PCRs (n = 667), 11 (1.7%) were positive, 654 (98.0%) were negative, and two were indeterminate (0.3%). Both of these infants subsequently tested negative at 6.1 and eight weeks respectively. Eleven (0.7%) infants were identified as HIV infected at the six‐ to ten‐week time‐point, 1553 (98.4%) tested negative and 15 (0.9%) indeterminate. Of the latter, all but one for whom no further results were found were negative on later testing. Five of the 11 infants first identified as HIV‐infected at six to ten weeks had had a negative birth HIV‐PCR indicating intrapartum or early postnatal transmission.

Seven of 450 infants (negative/unknown status) who had HIV‐PCR after 14 weeks old were HIV‐infected giving a transmission rate of 1.6% at this time‐point (median age at diagnosis 51.2 weeks (IQR 32.0 to 64.1)). Two infants had negative birth and six‐ to ten‐week HIV‐PCR results, four had negative six‐ to ten‐week results (no birth test) and the remaining infant was testing for the first time after fourteen weeks old.

### 3.5 Predictors of six‐week HIV‐PCR testing

Maternal characteristics associated with transmission risk (Tables 1 and 5) were also associated with not receiving a six‐ to ten‐week HIV‐PCR in both univariable and multivariable analyses. Infants who received EID at birth were 82% less likely to receive a follow‐up HIV‐PCR than those who did not, after adjusting for policy period, low birthweight and prematurity, and maternal characteristics associated with not undergoing a six‐ to ten‐week HIV‐PCR (aOR 0.18 (95% CI 0.12 to 0.26))(Table 5).

**Table 5:** Univariate and Multivariate analysis of predictors of having a follow‐up HIV‐PCR as per guidelines (i.e. 4 to 14 weeks old)

	Six to ten week PCR done ever	
	Crude OR (95% CI)	AOR (95% CI)
Maternal age >35 years	1.47 (0.99 to 2.16)	1.32 (0.84 to 2.10)
HIV diagnosis during pregnancy	0.71 (0.41 to 1.21)	1.10 (0.56 to 2.15)
Antenatal care – none	0.24 (0.15 to 0. 39)	0.25 (0.15 to 0.42)
Seroconversion during pregnancy	0.24 (0.11 to 0.52)	0.33 (0.13 to 0.88)
Duration of art ART<12w	0.45 (0.33 to 0.61)	0.61 (0.42 to 0.88)
Viral load>1000 copies/mL	0.51 (0.34 to 0.76)	0.68 (0.43 to 1.10)
Gestational age <37 weeks	0.56 (0.33 to 0.95)	1.12 (0.59 to 2.11)
Birthweight<2500 g	0.48 (0.33 to 0.69)	0.91 (0.56 to 1.47)
EID period 2 (vs. period 1)	0.63 (0.37 to 1.10)	0.92 (0.48 to 1.75)
EID period 3 (vs. period 1)	0.31 (0.18 to 0.55)	0.61 (0.30 to 1.47)
Birth HIV‐PCR present	0.15 (0.11 to 0.20)	0.18 (0.12 to 0.26)

ART, antiretroviral therapy; EID, early infant diagnosis.

## 4 Discussion

To the best of our knowledge this is the first study using longitudinal data to demonstrate the real‐world implementation of the progressive introduction of birth HIV‐PCR to an EID programme in a routine setting. The majority of infants (89%) received at least one HIV‐PCR, most around six weeks old. As the indications for birth HIV‐PCR expanded, the proportion receiving birth EID and more than one test increased and the median age at testing decreased, but there was no change in the proportion of infants receiving at least one HIV‐PCR, indicating that further improvement in EID uptake is needed. In addition, receipt of birth HIV‐PCR significantly reduced follow‐up testing. While coverage of birth HIV‐PCR during period 3 (indicated in all HIV‐exposed infants) was high overall (67%), it was considerably lower at the primary care facility (~35%) where >40% of deliveries occurred in this cohort (Table 3). This suggests that even in a relatively well‐resourced setting like the Western Cape, implementation of birth EID guidelines is challenging in primary care, the setting where most facility‐based deliveries are likely to occur in sub‐Saharan Africa.

Data presented in a review of the first year of the SA National EID Programme demonstrated an increase in birth EID coverage from 39% (high‐risk) to 93% within 12 months of the national guideline change to universal birth testing with a corresponding drop in six‐week testing (to 19%); the increase in ten‐week tests was modest 13. In our study, the majority of all birth testing occurred in infants born in hospital. During the first two policy periods, this could be expected owing to the definition of certain high‐risk criteria (prematurity, low birthweight). This is reflected in an analysis of Western Cape laboratory data in which, while the number of facilities offering birth EID increased during period 2 (high‐risk indication), the majority of tests continued to be performed in hospitals: 67% versus 33% sent from primary care 14. The national programme data 13 were not stratified by level of care and there are still limited data on how the EID recommendations are implemented in primary care facilities and in rural areas across SA.

### 4.1 Risk factors for vertical transmission

Almost half of all infants presented with at least one high‐risk factor for vertical transmission, yet only 30.8% received birth HIV‐PCR during period 2, the majority of those being premature and low birthweight infants (i.e. likely to be born in hospital). Although the likelihood of receiving a birth test increased with increasing number of risk factors, many high‐risk infants were missed. In a recently published EID study in Botswana, 16% of the cohort presented with maternal high‐risk criteria and all *in utero* transmission could be ascribed to receipt of <8 weeks of antenatal ART or known lack of viral suppression 15.

Nevertheless, our data demonstrate the maturation of the HIV treatment programme in Cape Town and some progress towards addressing vertical transmission risk factors: over 28 months an increasing number of women entered ANC known to be HIV‐infected, had conceived on ART and were on ART for longer durations before delivery.

### 4.2 HIV transmission and follow‐up testing

The number of HIV‐infected infants in the cohort was low (n = 32) so data need to be interpreted with caution. Overall vertical HIV transmission rate was 1.6%. This compares with a national rate of 1.4% at six weeks in 2016 down from 2.4% in 2012 16, 17. *In utero* transmission was 1.7% on average, highest during periods 1 (3.6%) and 2 (2.3%) when a sample of high‐risk infants was selected. A hospital‐based retrospective cohort study in Cape Town found a similar *in utero* transmission rate of 3.8% 18, and the Botswana cohort 3.3% 15 both under programmes of targeted birth EID. When the sample of exposed infants tested at birth is expanded to include those at low risk of infection, a reduced transmission rate would be expected. The average *in utero* transmission rate during the first year of the SA national birth EID programme was 1.1%; when stratified by province it was 2.6% in the Western Cape but the interval overlapped both period 2 and period 3 suggesting that the sample may have been biased towards high‐risk infants and is closer to that in period 2 13. The discrepancy may also be due to the maternal HIV prevalence in our study sample (14.7% at delivery) versus 18.9% for the whole province 19. In a Johannesburg hospital sample *in utero* transmission rate was 1.4% during period 3 20.

The detection rate between birth and six to ten weeks was lower than at birth (1%) except in period 3; this compares with rates of 0.4 to 0.5% in a hospital cohort during the same period of targeted birth testing 18. During period 3 there were no positive birth HIV‐PCR results and the detection rate between birth and six to ten weeks was 2.4% (vs. national rates of 1.4% at six weeks 13.) Almost half of these infants had a negative birth test indicating late intrapartum or early postnatal transmission and reinforcing the necessity of follow‐up testing.

Our study provides additional evidence that receipt of birth HIV‐PCR decreased the presentation for follow‐up testing 13, 14, 18. Receipt of birth EID reduced the likelihood of subsequent HIV testing after six weeks by 82%, even when controlling for other risk factors. This concurs with a retrospective analysis of a Cape Town hospital sample in which infants who had birth HIV‐PCR were 40% less likely to present for follow‐up testing 18; when follow‐up did occur in the birth cohort it was at older ages than among those without birth EID (8.6 vs. 7.1 weeks). We found that age at subsequent HIV‐PCR was greatest during the period of maximum birth testing, that is, 8.6 weeks in period 3; appropriate as the time‐point for testing had shifted from six to ten weeks. In a sensitivity analysis to assess the influence of birth EID on the likelihood of *ever* having a repeat HIV‐PCR after four weeks old (i.e. not restricted to the six‐ to ten‐week window), the direction of the associations remained unchanged. These findings are concerning given that the modelling data that supported the introduction of birth EID in SA demonstrated a loss of effectiveness if return for follow‐up testing dropped below 63% 5. In addition, at least 11 HIV‐infected infants in this cohort (35.5%) were late intrapartum or postnatally infected, emphasising the need for testing after the birth time‐point.

It has been suggested that receipt of a negative HIV‐PCR result at birth may provide false assurance to carers who then feel additional testing is not required 18. High‐risk criteria for HIV transmission were also associated with reduced follow‐up testing (Table 5). It is possible that these reflect poor health‐seeking behaviour in a group of women (for whatever reasons), of which failure to present for follow‐up infant testing is an additional example. The data presented here do not permit further speculation. In addition, the fragmentation of the South African health systems and the mobility of the population undermine the ability of health workers to identify HIV‐exposed infants for HIV‐PCR (e.g. when they present for routine immunization.) In contrast to the SA data, interim analysis of women in Lesotho indicated that the majority of women who received very early EID (0 to fourteen days) returned for results and follow‐up testing at six weeks 21, 22.

The proportion of indeterminate results was low at all time‐points, but accounted for 15.4% and 55.6% of non‐negative results at birth and six to ten weeks respectively. Similar results were reported in a review of birth EID in Johannesburg, 0.4% of all results, 24% of non‐negative results were indeterminate 23. In the Johannesburg study, additional resources were required to trace and retest these neonates. In our cohort all the infants were negative on repeat testing, in contrast to both the Johannesburg study and to a laboratory‐based cohort which demonstrated increased likelihood that infants with indeterminate HIV‐PCR result would be positive on subsequent testing in a setting of intensified VTP regimens 23, 24. It is possible that the subsequent negative HIV‐PCR results in our report were false negatives in the face of prolonged infant post‐exposure prophylaxis and/or exposure to ART in breast‐milk. Unfortunately, the data in the e‐register (and alternative electronic sources) did not include reliable infant prescription information beyond birth, and so we are unable to determine adherence to infant postnatal prophylaxis and assess risk factors for transmission in the postnatal period.

### 4.3 Strengths and limitations

We present prospective individual longitudinal follow‐up on a cohort of HIV‐exposed infants born at a primary care facility as well as in hospital. The cohort has an advantage over the aggregate laboratory data 13, 14 in that HIV‐PCR results could be attributed to individual, linked mother‐infant pairs. One of the acknowledged challenges of using routine laboratory data for surveillance in SA is the lack of an unique patient identifier and the inability to accurately de‐duplicate data 13, 16. Although the Western Cape has made substantial progress with issuing unique identifiers to newborns, operational challenges remain, and our sample was limited to linked mother‐infant pairs, compromising numbers that could be included, especially in period 1. While our study is strengthened by demonstrating real‐world implementation of expanded EID guidelines, it is consequently also dependent on the quality of routine clinical sources and we were unable to account for missing data. It is possible that not all the relevant HIV‐PCRs were recorded as an infant could have numerous alternative identifiers; we were also unable to determine whether HIV‐PCR tests occurred outside the Western Cape. Some infants may have died before follow‐up testing; prematurity and low birthweight contribute to infant mortality independent of HIV infection. Similarly infants may have been too acutely unwell to undergo birth EID.

## 5 Conclusions

In this study, we assessed the impact of different birth EID strategies, that of targeted birth testing of high‐risk infants, and universal testing of all HIV‐exposed infants. Targeted birth EID identified more HIV‐infected infants early, some of whom may otherwise have died before six weeks. However, universal testing simplified the VTP guidelines and increased the proportion of infants tested at birth. While our data demonstrate an encouraging response to EID guideline changes, birth HIV‐PCR was not well implemented at primary care level. Birth EID also compromised follow‐up testing at six or ten weeks and this LTFU may negate the model‐predicted benefits of birth HIV‐PCR 5. Additional intervention is required to reduce risk factors for transmission, to expand birth EID at primary care and to improve follow‐up testing at the ten‐week time‐point in SA.

## Competing interests

The authors have no conflicts of interest to declare.

## Authors’ contributions

AB, MO, KS, MK and MAD conceptualised and designed the study, contributed to operational needs and clinical oversight when required and critically reviewed the manuscript. EK conducted the study, cleaned and analysed the data and prepared the manuscript. JE, VT and MAD contributed to data management and analysis and critically reviewed the manuscript. All approved the final draft.
